# Postoperative intestinal obstruction in patients with biliary atresia impedes biliary excretion and results in subsequent liver transplantation

**DOI:** 10.1007/s00383-020-04807-9

**Published:** 2021-01-03

**Authors:** Aitaro Takimoto, Wataru Sumida, Hizuru Amano, Chiyoe Shirota, Takahisa Tainaka, Kazuki Yokota, Satoshi Makita, Akihiro Yasui, Yoko Kanou, Akinari Hinoki, Hiroo Uchida

**Affiliations:** Department of Pediatric Surgery, Nagoya University Graduate School of Medicine, 65 Tsurumai, Showa, Nagoya, 466-8550 Japan

**Keywords:** Biliary atresia, Intestinal obstruction, Bile secretion, Portoenterostomy

## Abstract

**Purpose:**

This study aimed to investigate the negative effects of intestinal obstruction for jaundice-free native liver survival after Kasai portoenterostomy (PE) for biliary atresia (BA).

**Methods:**

We retrospectively reviewed the records of patients who underwent PE for BA between 2006 and 2019. We evaluated the postoperative morbidity of intestinal obstruction for up to 2 years after PE and the effects of intestinal obstruction on jaundice-free native liver survival. On the basis of their initial operation, patients were divided into open portoenterostomy (Open-PE) and laparoscopic portoenterostomy (Lap-PE) groups, and morbidity was compared.

**Results:**

Of the 87 patients reviewed, 6 (6.9%) patients developed postoperative intestinal obstruction and underwent surgery to relieve the obstruction. The morbidity of early postoperative intestinal obstruction was 1.68 per 10,000 person days. The jaundice-free native liver survival rate among patients who once achieved jaundice-free status after PE was significantly lower in the patients with intestinal obstruction compared to in those without intestinal obstruction (0% vs. 73.8%; RR = 3.81, *p* = 0.007). No significant differences were seen in postoperative intestinal obstructions between the Open-PE and Lap-PE groups (*p* = 0.242).

**Conclusions:**

Intestinal obstruction negatively impact jaundice-free native liver survival, even in patients who once achieved jaundice-free status after PE for BA.

## Introduction

Biliary atresia (BA) is the most severe liver disease in neonates and early infancy. Kasai portoenterostomy (PE) is the first-line treatment to facilitate bile drainage. If PE fails to drain the bile, liver transplantation is the only means of survival for patients with BA. In Japan, 60% of patients with BA achieve postoperative decreases in serum bilirubin levels at 1 year of age [1]. The anicteric transplant-free survival rates at 5 and 20 years are approximately 60% and 50%, respectively [2].

Cholangitis is the most common complication that occurs after PE, and recurrent attacks of cholangitis are a prognostic marker for rapid liver failure resulting in the requirement for early liver transplantation [3].

Intestinal obstruction is a common complication that occurs after abdominal surgery. Intestinal obstruction can cause congestion of the intestinal bacteria and increase intra-intestinal pressure. Congestion of intestinal bacteria can cause ascending cholangitis. Increased intra-intestinal pressure may affect the porta hepatis through the Roux-en-Y limb, and the high pressure may destroy the fragile biliary drainage system. Thus, postoperative intestinal obstruction can cause ascending cholangitis after PE and impede biliary excretion, which can result in liver failure.

To the best of our knowledge, there have been no studies on the morbidity and influence of postoperative intestinal obstruction on biliary secretion after PE for BA management. This study was conducted to determine the effects of postoperative intestinal obstruction on jaundice-free native liver survival after PE for BA within the first 2 years after surgical treatment for BA.

## Patients and methods

We retrospectively reviewed the records of patients who underwent PE for BA in our hospital between January 2006 and March 2019. The study was approved by the Ethics Committee (Ref No. 2020-0058).

First, we evaluated the postoperative morbidity and interventions associated with intestinal obstruction for up to 2 years after PE for BA. The observation period ended when the patient underwent transplantation or died within 2 years after PE. The effects of intestinal obstruction on jaundice clearance and the need for liver transplantation were investigated as short-term outcomes. Jaundice-free native liver survival was evaluated at the end of the observation period.

Next, patients were divided into two groups according to the initial operation they underwent: open portoenterostomy (Open-PE) and laparoscopic portoenterostomy (Lap-PE). We compared the postoperative incidence of intestinal obstruction between the two groups. Early postoperative management differed between the two groups. In the Open-PE group, most patients started ingestion on postoperative day (POD) 5, and steroids were administrated starting on POD 7. In the Lap-PE group, patients started ingestion on POD 3, and steroids were administrated starting on POD 5 in most cases. However, out-patient management, such as medication for choleretics and intestinal flora, was similar in both groups.

Data are expressed as the median and range. Statistical analysis was performed using a Fisher’s exact test for categorical variables and a Mann–Whitney *U* test for continuous variables. *P* < 0.05 was considered statistically significant.

## Results

We identified 87 patients who underwent PE during the study period. Among them, six patients (6.9%) developed intestinal obstruction within 2 years after the initial surgery for BA. The median (range) duration to early postoperative intestinal obstruction was 248 (9–593) days. The overall morbidity rate was 1.68 per 10,000 person days. No significant differences were observed in terms of the sex, age at first operation, type of BA, initial PE factors (Open-PE or Lap-PE, blood loss, operation time), and revision history between patients with and without intestinal obstruction (Table 1). The jaundice-free rate at the end of the observation period was significantly lower in patients with intestinal obstruction compared to in those without intestinal obstruction [0% (0/6) vs. 55.6% (45/81), respectively; RR = 2.25, *p* = 0.010]. All six patients with postoperative intestinal obstruction required liver transplantation due to jaundice (100% vs. 39.5%; RR = 2.53, p = 0.006). Next, we focused on the patients who achieved jaundice-free status at least once. There were 65 patients whose jaundice was resolved after PE. Twenty-two patients including 2 ileus patients, who never achieved jaundice-free status after PE, were excluded from the comparative study. Four out of six patients had once achieved jaundice-free status after PE before intestinal obstruction. However, those four patients all experienced jaundice recurrence after intestinal obstruction, and required liver transplantation. Among the 65 patients whose jaundice was resolved by PE, the jaundice-free native liver survival rate at the end of the observation period was significantly lower in those with intestinal obstruction than in those without intestinal obstruction [0/4 (0%) vs. 45/61 (73.8%), respectively; RR = 3.81, *p* = 0.007].

**Table 1:** Comparison of patient characteristics variables between the patients with/without intestinal obstruction

	Patients with IO	Patients without IO	*p* value
*n*	6 (6.9%)	81 (93.1%)	
Female	4 (66.7%)	54 (66.7%)	1
Age of first operation	62 (45–95)	62 (28–144)	0.834
Type of BA I/II/III	0/0/6	6/0/75	1
*The initial operation*			
Open-PE/Lap-PE	2/4	46/35	0.401
Blood loss(g)	30 (6–35)	39 (3–367)	0.304
Time (min)	289 (209–458)	285 (167–497)	0.627
Revision	0 (0%)	23 (28.4%)	0.334
Jaundice disappearance rate after PE	4/6 (66.7%)	61/81 (75.3%)	0.640
Jaundice-free native liver survival rate among patients who had once achieved jaundice-free status	0/4 (0%)	45/61 (73.8%)	0.007
Requirement of liver transplantation	6/6 (100%)	32/81 (39.5%)	0.006

*PE* portoenterostomy, *IO* intestinal obstruction

Values are presented as *n* (%) or median (range)

We reviewed the records of patients with intestinal obstruction after PE (Table 2). All six patients needed surgery for intestinal obstruction. Two patients underwent operation 2 or 4 days after a nasogastric tube was placed, whereas the other four patients underwent emergency operation. In all patients, the cause of obstruction was related to the Roux-en-Y limb. Four patients had a fibrous band near the Roux-en-Y anastomotic site, and one patient underwent intestinal resection because of an internal hernia constructed with the ascending jejunal limb, which adhered to the liver biopsy scar.

**Table 2:** Characteristics of the patients with intestinal obstruction after portoenterostomy

Age(days)/sex	Open-PE or Lap-PE	Postoperative jaundice	Onset of IO (POD)	Treatment	Operative findings	Procedure	Cholangitis before IO	Cholangitis after IO	Reasons of LTx	Age of LTx
56/F	Open	Anicteric	308	Emergency operation	Band of fibrous tissue	Cut of the band	None	Recurrent	Multiple bile lakes Cholangitis jaundice	478
95/F	Open	Remain	9	Emergency operation	Adhesion to the liver biopsy site, internal hernia	Intestinal resection	None	None	Jaundice	Dead while waiting for LTx
45/F	Lap	Remain	15	NG tube → Operation	Adhesion near the R-Y anastomotic site	Adhesiolysis	None	None	Jaundice	242
80/M	Lap	Anicteric	593	Emergency operation	Band of fibrous tissue	Cut of the band	2	1	Jaundice	832
66/M	Lap	Anicteric	222	Emergency operation	Band of fibrous tissue	Cut of the band	2	1	Jaundice	Waiting for LTx
57/F	Lap	Anicteric	340	NG tube → Operation	Band of fibrous tissue	Cut of the band	4	Recurrent	Multiple bile lakes Cholangitis jaundice	370

*IO* intestinal obstruction, *NG tube* nasogastric tube

Two patients with intestinal obstruction on POD 9 and POD 15 showed no jaundice-free status after PE, and one underwent liver transplantation within 1 year after PE. The other patient, who had congenital heart disease, died due to intracranial hemorrhage 6 months after a radical cardiac operation while waiting for a liver transplant. The other four patients achieved jaundice-free status with their native liver after PE. However, jaundice recurred after the operation for intestinal obstruction, resulting in a requirement for liver transplantation. One patient with jaundice continues to wait for a liver transplant at the time of writing.

**Fig. 1:**
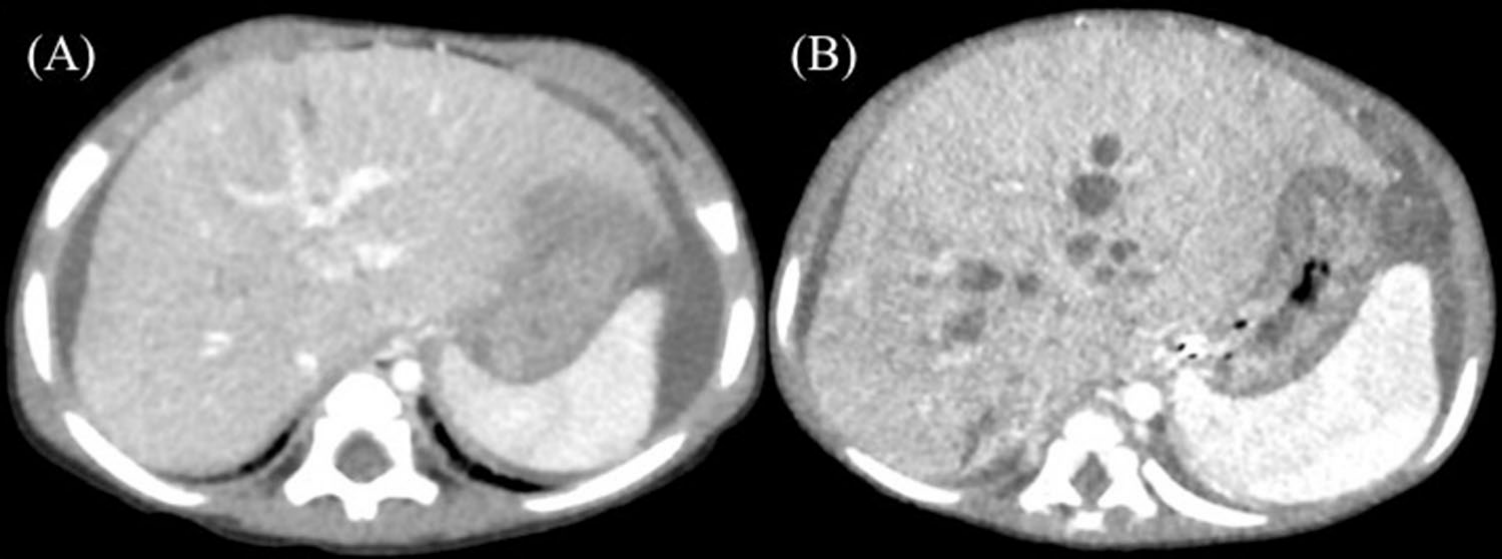
Enhanced computed tomography after portoenterostomy. (A) Before intestinal obstruction. (B) After intestinal obstruction. Multiple bile lakes suddenly appear throughout the liver

Two patients who developed intestinal obstruction soon after PE experienced no cholangitis until liver transplantation. Two patients experienced several attacks of cholangitis before and after intestinal obstruction. Two patients with multiple bile lakes after intestinal obstruction had prolonged fever due to cholangitis; these patients had no history of bile lakes before intestinal obstruction (Fig. 1).

Patients were stratified on the basis of the type of operation: Open-PE (*n* = 48) and Lap-PE (*n* = 39) (Table 3). Of the six patients with postoperative intestinal obstruction, two were from the Open-PE group (4.2%; 2/48), while the remaining four patients were from the Lap-PE group (10.3%; 4/39). No significant difference was seen in the morbidity rate between the Open-PE and Lap-PE groups (0.97 vs. 2.67 per 10,000 person days, respectively; *p* = 0.242).

**Table 3:** Comparison of patient characteristics variables between the Open-PE group and Lap-PE group

	Open-PE	Lap-PE	*p* value
*n*	48 (55.2%)	39 (44.8%)	
Female	29 (60%)	29 (74%)	0.17
Age of first operation	74 (35–144)	55 (28–89)	< 0.05
Revision	11 (22.9%)	12 (30.8%)	0.409
Postoperative IO	2 (4.2%)	4 (10.3%)	0.265
IO morbidity (/10,000 person days)	0.97	2.67	0.242
Liver transplantation	18 (37.5%)	18 (46.2%)	0.512
Jaundice free at the end of observation	28 (58%)	17 (44%)	0.171

*PE*: portoenterostomy, *IO*: intestinal obstruction

Values are presented as *n* (%) or median (range)

## Discussion

Previous studies have reported an incidence rate of intestinal obstruction after Open-PE for BA of approximately 3–8% [4, 5], which was similar to our study (6.9%, 6/87 patients; 4.2%, 2/48 Open-PE group; 10.3%; 4/39 Lap-PE group). In the present study, all six patients with intestinal obstruction had persistent jaundice and eventually required liver transplantation. The jaundice-free native liver survival differed significantly between patients with and without intestinal obstruction. However, no significant difference was observed in patient characteristics that impacted the need for liver transplantation between patients with and without intestinal obstruction [6, 7]. Thus, the present study considered postoperative intestinal obstruction as a novel etiopathology that causes jaundice secondary to biliary obstruction. Bile secretion from the hepatic hilum after PE depends on a fine and fragile structure similar to the bile duct [8]. Therefore, this fine fragile “pseudo-bile duct” may have been damaged due to an increase in intra-intestinal pressure and bacterial translocation caused by postoperative intestinal obstruction. Two patients with early postoperative intestinal obstruction never achieved jaundice-free status. Therefore, in those two patients, it was difficult to affirm that liver transplantation was caused by intestinal obstruction. On the other hand, four patients who became anicteric after the initial operation subsequently developed recurrent jaundice after intestinal obstruction. Among the 65 patients whose jaundice was resolved by PE, the jaundice-free native liver survival rate at the end of the observation period was significantly lower in patients with intestinal obstruction than in patients without intestinal obstruction. Therefore, the findings suggest that intestinal obstruction independently has a negative impact on biliary secretion, resulting in recurrent jaundice and subsequent liver transplantation.

In all six patients, the formation of fibrous bands or adhesions near the Roux-en-Y anastomotic site caused intestinal obstruction. The internal pressure in the ascending jejunal limb may have readily increased because the origin of the obstruction was close to the Roux-en-Y anastomotic site. Laparoscopy is reported to be associated with a significant and sustained decrease in the rate of intestinal obstruction [9]. However, there are no comparative reports of intestinal obstruction between Lap-PE and Open-PE. In our review, we found no significant difference in the incidence of intestinal obstruction between patients who underwent laparotomy or laparoscopic surgery, which was contrary to our hypothesis. As part of the standard institutional protocol at our hospital, Roux-en-Y anastomosis is created outside the umbilical incision during Lap-PE. Therefore, the risk of fibrous adhesion formation around the Roux-en-Y anastomotic site is comparable between laparotomy and laparoscopic surgery. Four of the six patients who underwent emergency operation for intestinal obstruction developed recurrent jaundice. Even short-duration intestinal obstructions that cause high intra-intestinal pressure could affect bile secretion, resulting in recurrent jaundice.

In four of the six patients with intestinal obstruction, postoperative cholangitis was not responsible for subsequent liver transplantation. In the present study, it is uncertain whether intestinal obstruction caused cholangitis. However, two patients revealed multiple bile lakes after intestinal obstruction, indicating that intestinal obstruction could result in the obstruction of biliary secretion from the “pseudo-bile duct” at the hepatic hilum.

Honna et al. [10] reported that in 1 of the 41 patients who underwent the replacement of the antireflux valve, the Nakajo antireflux valve prevented the incidence of cholangitis in conditions such as intestinal obstruction. Creating an antireflux valve at the Roux-en-Y anastomotic site may be useful to prevent an increase in intra-intestinal pressure within the ascending jejunal limb and maintain bile secretion. However, there is no clear evidence that an antireflux valve prevents cholangitis in clinical settings. A single-center study that evaluated the routine implementation of an antireflux valve reported a high rate of anicteric transplant-free survival [11]. Therefore, it is necessary to prospectively investigate whether an antireflux valve prevents an increase in the internal pressure of the hepatic hilum whenever the intra-intestinal pressure is elevated and whether this valve helps prevent ascending cholangitis.

Our study had some limitations, including the retrospective design and small number of patients. Further prospective studies with more patients are required to validate the effect of postoperative intestinal obstruction on the outcome of patients with BA.

## Conclusion

Intestinal obstruction following PE for BA can result in persistent jaundice and reduce jaundice-free native liver survival. Intestinal obstruction can independently cause recurrent jaundice and subsequent liver transplantation for the patients who once achieved jaundice-free after PE for BA. Further, there is no significant difference in morbidity of intestinal obstruction between patients who undergo open or laparoscopic portoenterostomy.
